# Controlled Crystallization of Nanocrystalline Apatite via Vapor Diffusion on Bacterial Cellulose Membranes Obtained from Mango Waste

**DOI:** 10.1021/acs.cgd.5c00437

**Published:** 2025-08-08

**Authors:** Isabel Navarro-Zabarburú, Amparo Iris Zavaleta, Susana Calderón-Toledo, Jaime Gómez-Morales, Pedro Alvarez-Lloret

**Affiliations:** † Laboratory of Molecular Biology, Faculty of Pharmacy and Biochemistry, 33209 Universidad Nacional Mayor de San Marcos, Lima 506, Peru; ‡ Laboratory of Crystallographic Studies, IACT-CSIC, Avda. Las Palmeras 4, E-18100 Armilla, Spain; § Department of Geology, 16763 University of Oviedo, E-33005 Oviedo, Spain

## Abstract

Hybrid bacterial cellulose (BC)-calcium phosphate apatite (Ap) composite was successfully synthesized via the sitting drop vapor diffusion crystallization method. The BC matrix was produced using the bacterial strain *Komagataeibacter* sp. SU12 cultured in a medium derived from mango juice waste, underscoring a sustainable strategy for biopolymer production. The resulting BC-Ap composite exhibited plate-like apatite crystals, as confirmed by X-ray diffraction analyses, which were heterogeneously distributed on the BC matrix and coupled to the nanocellulose surface fibers. An increase in mineral content in the BC-Ap composites over the experimental reaction times (1–15 days) was observed by thermogravimetry analyses. Spectroscopic analyses confirmed the presence of characteristic BC functional groups (e.g., hydroxyl and carboxylate), and the vibrational modes associated with phosphate (ν_1_–ν_4_ of PO_4_^3–^), corroborating the formation of apatite within the BC–Ap material. These findings suggest that the vapor diffusion crystallization method is an effective approach for the controlled mineralization of BC nanofibers with nanocrystalline apatite, yielding a bioinspired material with promising potential application in bone tissue engineering. Additionally, the use of mango-processing waste as a carbon source for BC production offers a sustainable and cost-efficient alternative, supporting the advancement of green technology and biocompatible routes for material design.

## Graphical abstract

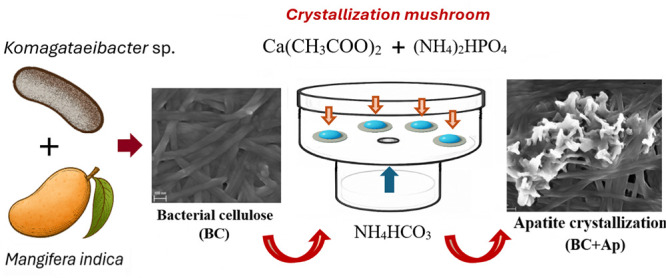

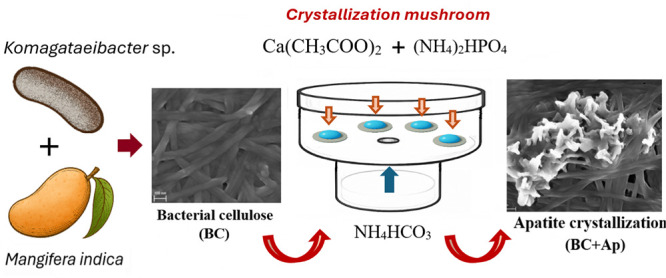

## 1 Introduction

Bacterial cellulose (BC) is a biopolymer characterized by its excellent biocompatibility, high mechanical strength, and exceptional water retention capability.^1–4^ BC can be synthesized by a variety of bacteria, including Gram-negative (v.gr. genus *Salmonella, Agrobacterium, Alcaligenes, Azotobacter, Pseudomonas*, and *Rhizobium*), and Gram-positive () bacteria.^5,6^ The genus *Komagataeibacter* is known for efficiently producing bacterial nanocellulose from carbon-rich sources, yielding BC matrices with consistent physicochemical, morphological, and mechanical characteristics.^7,8^ The Hestrin-Schramm medium is the most suitable standard for cultivating BC, despite being more expensive than other more accessible sources. The use of natural medium derived from plant product waste has been proposed as an effective alternative to increase the viability of BC production.^9–12^

Hydroxyapatite (HAp; Ca_10_(PO_4_)_6_(OH)_2_) crystallization holds significant importance in several biomineralization processes and biomaterial engineering. HAp is the most abundant inorganic component of mineralized tissues (e.g., bone and teeth). In bone, the HAp shows distinctive structural features, comprising nanoscale platelets interwoven with an organic matrix composed mainly of collagen, noncollagenous proteins, and small organic molecules such as citrate.^13–15^ To resemble bone or tooth structures, synthetic apatite should replicate the morphological and crystalline properties of biological HAp, evolving nano or micrometer-sized crystals, and proper orientation.^16,17^ Moreover, the chemical composition should also reflect the Ca/P ratios of the biological HAp incorporating CO_3_^2–^ that partially replace OH^–^ and PO_4_^3–^ groups, and metal ions such as Mg^2+^ in atomic positions of Ca^2+^ in the case of bone HAp.^18^ In addition, the chemical stability of synthetic apatite-based materials at different pH levels and temperature ranges is crucial, along with porosity and density, to facilitate tissue integration.^19^

Hybrid materials composed of BC-HAp have demonstrated potential capabilities for several industrial and technological applications.^20^ Previous research has investigated the use of BC-HAp scaffolds for various biomedical applications, including wound dressing, tissue engineering, vascular stents, and bone grafts.^1,21,22^ This biohybrid material offers high biocompatibility with calcified tissues, stability under physiological conditions, and an enhanced ability to stimulate osseointegration. Recent studies have shown the effectiveness of the BC-HAp composites in supporting bone cell adhesion, proliferation, and migration, which are crucial features for bone healing and regeneration.^23,24^ On the other hand, for successful tissue integration, these BC-HAp composites must exhibit appropriate biodegradability, porosity and structure to facilitate cellular infiltration and nutrient exchange.^25,26^ Additionally, these materials must also provide sufficient mechanical strength to withstand specific physiological loads associated with each tissue function.^25,27,28^ Such functionalized biopolymer-apatite composites have demonstrated significant potential in guided bone regeneration, serving as scaffolds or membranes that facilitate bone formation while preventing soft tissue infiltration.^29^

The synthesis of BC-HAp materials has been previously explored using several crystallization methods. A biomimetic route proposes the precipitation of HAp by immersing BC in simulated body fluid (SBF) solutions.^30,31^ Other wet methods promote HAp crystal formation in BC surfaces through chemical reactions using Ca/P-rich solutions.^32,33^ In all of them, a key limitation is controlling mineral precipitation, which has led to the development of chemical modifications of BC fibers with anionic and water-soluble polymers (e.g., polyvinylpyrrolidone or carboxymethyl cellulose) and the adjustment of specific reaction conditions (i.e., pH, aging time, temperature).^34^ The sitting drop vapor diffusion (SDVD) crystallization method has previously been employed to synthesize low-crystalline, plate-like apatite crystals that resemble the properties of biological nanocrystalline HAp.^35^ This methodology enables crystallization on heteronucleant surfaces with high reproducibility under controlled experimental conditions in small microliter reaction volumes. While the SDVD procedure has been used in previous studies to achieve heterogeneous nucleation of HAp on various mineral and organic surfaces,^36–38^ its application to the BC surface has not yet been explored. The main advantage of SDVD over the above-reported immersion methods^30–33^ is that it allows coating only one of the two layers of a substrate, either the outer or the inner layer. In contrast, immersion methods typically coat both surfaces with significantly lower control over precipitate formation. This SDVD crystallization method can be exploited to create bifunctional membranes, with osteoinductive and barrier functions on opposite sides. This dual functionality has potential applications in (guided) bone regeneration, as previously reported for eggshell membrane.^38,39^

Synthetic polymers are widely used to provide versatility and strength to materials. However, these polymers have significant drawbacks, including substantial environmental impact and the release of toxic substances during production and usage.^40^ In contrast, cellulose-mineral composites are gaining popularity in various industries due to their distinctive properties, such as nontoxicity, high biocompatibility, reduced environmental footprint, and potential long-term cost reduction.^41^ For example, cellulose-silica composites combine the biodegradability and flexibility of organic cellulose with the thermal stability and mechanical strength of inorganic silica,^42,43^ making them ideal for various applications like thermal and acoustic insulation.^44^ Other materials, such as cellulose-zeolite composite, exhibit enhanced biodegradability and mechanical strength due to the organic substrate features, while zeolite improves metal removal efficiency through its high adsorption and ion exchange capacities.^45^ These overall properties lead to improved performance in advanced filtration systems and technological applications requiring high removal effectiveness.

This study explores the use of bacterial cellulose (BC) as an organic template for the controlled precipitation of calcium phosphate (CaP) using the SDVD crystallization method. For this purpose, BC membranes were synthesized from *Komagataeibacter* sp. SU12 (bacterial strain isolated from kombucha tea), utilizing plant waste extract from as a culture medium (i.e., carbon-rich source). This approach may help reduce environmental impact by recycling agricultural waste generated during food production, while also establishing biocompatible pathways for novel material design. Thus, the current study has two main objectives: (1) to evaluate the effectiveness of the SDVD crystallization method in controlling mineral deposition on bacterial cellulose nanofibers, and (2) to characterize the key physicochemical, morphological, and microstructural properties of the resulting BC–CaP composite, with potential relevance for different applications, such as guided bone regeneration.

## 2 Materials and Methods

### 2.1 Bacterial Cellulose (BC) Production

The bacterial cellulose (BC) was synthesized using the *Komagataeibacter* sp. SU12 strain, isolated from kombucha tea at the Molecular Biology Laboratory, Faculty of Pharmacy and Biochemistry, National University of San Marcos (Lima, Peru). The mango pulp ( var. Edward) was processed using an electric blender, and centrifugation at 45 Relative Centrifugal Force (RCF) and 25 °C for 10 min. The supernatant, referred to as mango extract (ME), was collected and autoclaved at 121 °C for 3 min at 15 psi. Reducing sugar concentration in the ME was quantified using the 3,5-dinitrosalicylic acid assay,^46^ with a glucose standard curve (*R* ^2^ = 0.99). The total reducing sugar concentration in the ME was adjusted to 2.0% (w/v) for use as a carbon source in the cultivation medium. The ME medium also contained 0.5% yeast extract (Oxoid, UK), 0.5% peptone (Gibco, USA), 0.27% Na_2_HPO_4_ (J.T. Baker, Mexico), and 0.115% citric acid (Amresco, USA). The pH of the medium was adjusted to 6.0 using 0.1 M NaOH solution and was subsequently autoclaved at 121 °C for 15 min at 15 psi. Reactivation of *Komagataeibacter* sp. SU12 was carried out by transferring a colony to HS medium^47^ prepared in 250 mL flasks containing 50 mL of medium. The strain was incubated at 2 RCF and 30 °C for 48 h.^48,49^ Subsequently, 10% (v/v) of the reactivated culture was inoculated into 500 mL of ME medium in a 2 L flask. Cultivation was conducted under static conditions at 30 °C for 7 days. The BC film formed at the air-medium interface was harvested, immersed in a 0.1 M NaOH solution (pH ≈ 13), and incubated at 50 °C for 6 h to remove impurities. The BC was then washed with distilled water until a neutral pH was achieved. Finally, the purified BC film was dried at 40 °C for 24 h and stored in a moisture-free environment until used in crystallization experiments.

### 2.2 Crystallization Experiments

BC round pieces (Ø 8 mm) were sectioned from the previously obtained film using a biopsy punch (Kai Medical, Japan). The SDVD crystallization experiments were performed in “crystallization mushrooms” (Triana Sci. & Tech., Spain), which consist of a glass microreactor device with two cylindrical glass chambers connected through a Ø 6 mm hole. The BC pieces were placed in the upper chamber and 40 μL droplets, consisting of 20 μL of 50 mM Ca­(CH_3_COO)_2_ and 20 μL of 30 mM (NH_4_)_2_HPO_4_ solutions, were deposited on them covering the full surfaces. The Ca/P ratio in the mixed solutions was set at 5:3 (∼1.67). Then, 3 mL of 40 mM NH_4_HCO_3_ solution was deposited in the lower chamber (reservoir) and the mushrooms were sealed with a top cap using high-vacuum silicone grease (Dow Corning, USA). The concentration of reagents was chosen based on previous work.^35^ The pH was measured with a pH probe (Titan model, Sentron, The Netherlands). The crystallization experiments were conducted at 1, 3, 5, 7, and 15-days, at ambient conditions (room temperature ∼25 °C and ∼1 atm). All reagents were supplied by Sigma-Aldrich (>99.00% pure, St. Louis, MO, USA), and solutions were prepared using ultrapure Milli-Q water (0.22 μS, 25 °C, Millipore, Burlington, MA, USA).

### 2.3 Characterization of Mineralized Bacterial Cellulose (BC-Ap)

For scanning electron microscopy (SEM) observations, samples were Au-coated (Balzers SCD 004) and examined using Quanta 650F and JEOL JSM-5600 microscopes. Semiquantitative chemical analysis was performed by energy-dispersive X-ray microanalysis (EDX) using a Bruker xFlash 6/30 detector. The acceleration voltage was set between 5 and 20 kV for the image acquisition. High-resolution images (HR-SEM) were acquired using a field-emission SEM microscope (FESEM) Zeiss Auriga (Carl Zeiss SMT Jena, Germany) operated at an acceleration voltage of 3 kV, high vacuum (∼10^–4^ mbar), a working distance of 4 mm, and an aperture of 30 μm. All samples for HR-SEM observations were previously Au-coated (Emitech K975X). The crystal dimensions (length and width) were obtained from SEM images using the ImageJ software.

Two-dimensional X-ray diffraction (2D-XRD) patterns were acquired using an X-ray diffractometer (Bruker D8 DISCOVER; Billerica, MA, USA) equipped with an area detector (DECTRIS PILATUS 3100 K-A; Baden, Switzerland). The XRD experimental conditions were CuKα (λ = 1.5406 Å) radiation at 50 kV and 30 mA, with a pinhole diameter collimator of 0.5 mm. The 2D-XRD patterns were registered within the 2θ scanning angle range from 8 to 50°, considering 0.02° 2θ steps and 40 s/step. The intensities concentrated in arcs within the Debye diffraction rings (corresponding to specific *d*-spacings) were integrated to obtain a unidimensional scan (i.e., 2θ pattern).

Fourier-transform infrared (FTIR) spectroscopy analyses were conducted using a JASCO 6200 spectrometer (JASCO, Tokyo, Japan) equipped with an attenuated total reflectance (ATR) diamond crystal accessory. Spectra were collected at a resolution of 2 cm^–1^ with 124 accumulations over a spectral range of 400–4000 cm^–1^. Additionally, Raman spectroscopic characterization was performed with a JASCO NRS-5100 Micro-Raman spectrometer (JASCO, Tokyo, Japan) covering a spectral range of 300–1800 cm^–1^, with a 20 s exposure time and 5 scans accumulations, maintaining an average spectral resolution of 1.6 cm^–1^. The excitation was provided by a diode laser (λ_exc._ = 785 nm) coupled with a Peltier-cooled charge-coupled device (CCD: 1064 × 256 pixels dimension). The instrument was calibrated using a silicon standard (reference position at 520 cm^–1^) before data acquisition.

Thermogravimetric analyses (TGA-DSC3+, Mettler Toledo, Columbus, OH, USA) were performed under air between 25 and 900 °C at a heating rate of 10 °C/min. Three samples were analyzed for each experimental reaction time to determine the residual mass at the end of the runs.

## 3 Results and Discussion

### 3.1 Bacterial Cellulose (BC) Membrane Production

Bacterial cellulose (BC) exhibits several distinctive properties, including an ultrafine nanofiber network with a complex architecture, high mechanical strength, in situ moldability and flexibility, and chemical purity (free of lignin and hemicellulose), which differentiate it from other forms of plant cellulose. The genus *Komagataeibacter* used is particularly noted for its exceptional cellulose-producing capacity.^10^ However, the high cost of the carbon sources for cellulose production poses a significant challenge.^50^ The current study proposes a mango waste-based medium ( var. Edward) to address this issue. This strategy reduces production costs and utilizes a sustainable resource, contributing to a more cost-effective and environmentally friendly solution for BC manufacturing.

In our research, BC membranes were synthesized using the activity of *Komagataeibacter* sp. SU12, which forms at the air–liquid interface of the mango waste-based medium (Figure 1a). Static fermentation conditions facilitate efficient cellulose accumulation under aerobic culture conditions, resulting in biofilms (Figure 1b) with approximate thicknesses of 0.05 mm, manageable for the proposed crystallization procedure. The surface appearance of the raw BC obtained is shown in Figure 1c,d. The BC network is characterized by cellulose nanofibers with random orientation, converging either perpendicularly or parallel to each other (Figure 1c). These individual cellulose fibers have diameters ranging from 30 to 60 nm, assembled into cross-linked bundles reaching widths of 100 nm (Figure 1d). It is noteworthy that the nanomorphology of BC shows fibers approximately one hundred times finer than those found in plant cellulose,^51^ thus increasing its relative specific surface area. This structure, along with its high porosity, allows BC to retain significantly more water, providing high moisture resistance, elasticity, and adaptability-features that are optimal for the controlled crystallization experiments following performed.

**Figure 1:**
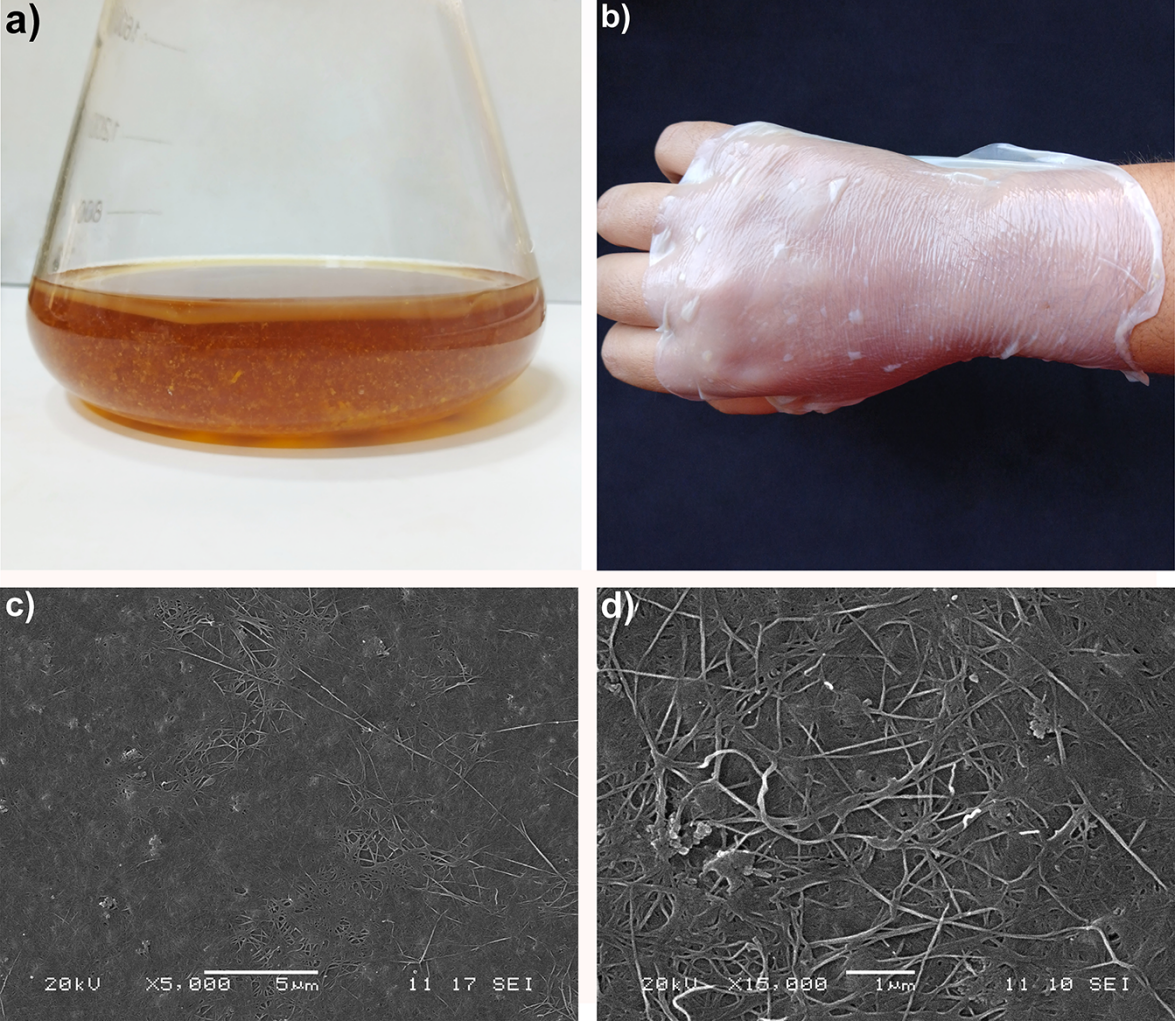
Production and morphology of BC derived from *Mangifera indica* extract medium using *Komagataeibacter* sp. SU12. (a) Laboratory production of BC in a static system, (b) Purified BC in thin membrane morphology, (c, d) SEM images showing the nanocellulose structure in the produced BC.

### 3.2 Vapor Diffusion Crystallization Process and Characterization of Mineralized BC Membranes

Previous studies have addressed apatite precipitation on BC membranes by immersing the material in a simulated body fluid (SBF) solution.^26,32,52,53^ It was shown that the chemical surface properties of BC are crucial as these features affect the organic–inorganic interactions that play a key role in the CaP precipitation.^52^ Additionally, BC-Ap hybrid composites have been created by incubating Ap particles in the BC production medium.^54^ Immersion methods do not allow for control over surface mineralization in the assembled biohybrid material, as these methods typically coat both sides of the BC membranes.^34^ In contrast, our findings demonstrate that the VDSD method facilitates the control of apatite coating on one side of the BC membrane, leaving the other side unmineralized, thus enabling the customization of some coating properties such as the amount of material precipitated and the extent of the coating on the mineralized BC surface and its nanofibers.

During the application of the VDSD method, the NH_3_ and CO_2_ vapors produced from the decomposition of the NH_4_HCO_3_ solution in the lower chamber of the *crystallization mushroom* (eqs 1 and 2), travel through a 6 mm hole from the lower to the upper chamber.^35^ This process increases the partial pressures of both gases in the upper chamber. These gases then diffuse into the microdroplets deposited on the outer side of the BC membranes. As NH_3_ diffuses through the droplets containing CaCH_3_COO^+^ and HPO_4_^2–^ ions, the pH of the microdroplet rises from approximately 4.8 to 8.5. This pH increase leads to the progressive decomposition of CaCH_3_COO^+^ complexes, releasing Ca^2+^ ions, while promotes the formation of PO_4_^3–^ according to eq 3, and CO_3_^2–^ ions, according to eqs 4–6, eventually increasing their concentration at the solid-solution interface, and then reaching the critical supersaturation necessary for heterogeneous nucleation of CaP. The local ion increase is favored by the presence of polar chemical surface groups of the cellulose.^55^ The overall precipitation process, which considers a CO_3_^2–^-Ap as an example of a solid phase, is illustrated in eq 7.

$${{\mathrm{NH}}_{4}}^{+}+{\mathrm{OH}}^{-}={\mathrm{NH}}_{3\left(\mathrm{aq}\right)}+H_{2}O={\mathrm{NH}}_{3\left(\mathrm{gas}\right)}$$ 1

$${{{\mathrm{HCO}}_{3}}^{-}}_{\left(\mathrm{aq}\right)}+H^{+}={\mathrm{CO}}_{2\left(\mathrm{aq}\right)}+H_{2}O={\mathrm{CO}}_{2\left(\mathrm{gas}\right)}$$ 2

$${{\mathrm{HPO}}_{4}}^{-}+{\mathrm{OH}}^{-}={{\mathrm{PO}}_{4}}^{3-}+H_{2}O$$ 3

$${\mathrm{CO}}_{2\left(\mathrm{gas}\right)}+H_{2}O=H_{2}{\mathrm{CO}}_{3}$$ 4

$$H_{2}{\mathrm{CO}}_{3}+{\mathrm{OH}}^{-}={{\mathrm{HCO}}_{3}}^{-}+H_{2}O$$ 5

$${{\mathrm{HCO}}_{3}}^{-}+{\mathrm{OH}}^{-}={{\mathrm{CO}}_{3}}^{2-}$$ 6

$$(5-x){\mathrm{Ca}}^{2+}+(3-x){{\mathrm{PO}}_{4}}^{-}+xC{O_{3}}^{2-}+{\mathrm{OH}}^{-}={\mathrm{Ca}}_{5}({\mathrm{PO}}_{4}{)}_{3-x}({\mathrm{CO}}_{3}{)}_{x}\mathrm{OH}$$ 7

When increasing the experimental time from 1 to 15 days, we observed a marked increase for mineral precipitated on the BC outer surfaces (Figure 2), due to the progressive release of Ca^2+^ ions from the Ca-CH_3_COO^–^ complexes. These complexes act as Ca reservoir during the whole period sustaining the precipitation. This crystal formation, which increases with aging time, occurs in a heterogeneous distribution on the BC surface in relation to the porous structure and permeation capacity of the pristine cellulose (Figure 2a). At early reaction times (1 and 3 days; Figure 2b,c), the CaP deposits appear highly dispersed, becoming more abundant and forming aggregates at longer reaction times (5 and 7 days; Figure 2d,e), and eventually coating almost the entire cellulose surface after 15 days (Figure 2f). The increasing mineralization with reaction time is further confirmed by the total amount of mineral content determined by thermogravimetric analyses (provided below). It should be noted that, at the end of the experiments the droplet volume remained practically unchanged, and no precipitation was observed on the inner side of the membranes.

**Figure 2:**
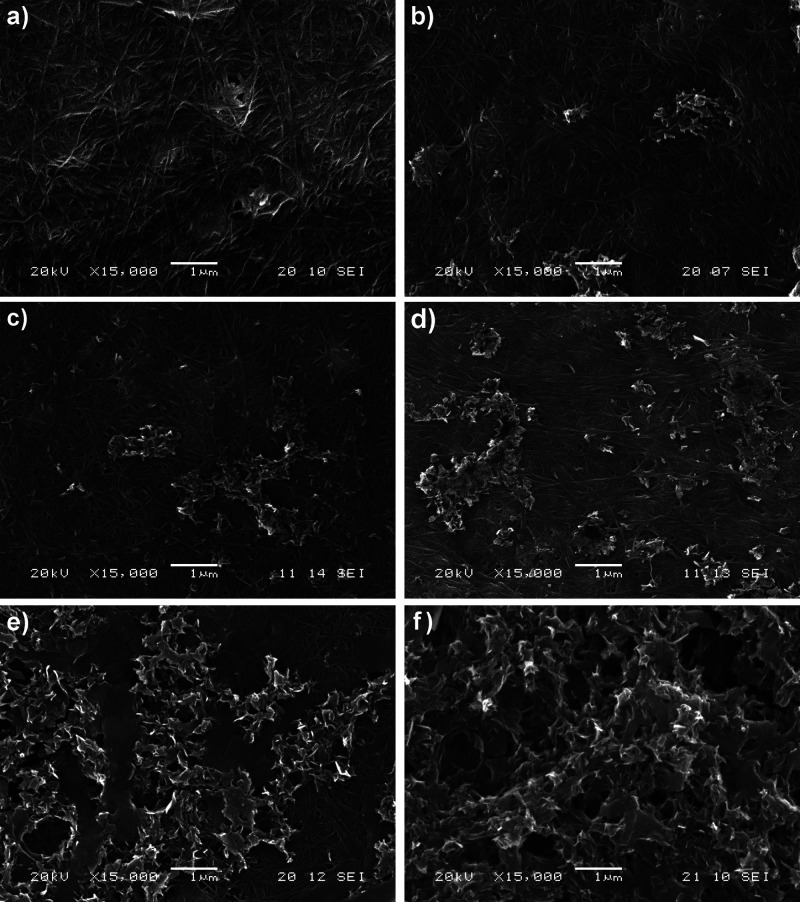
SEM images of CaP precipitation on BC surface for different reaction times. (a) Pristine BC surface; (b–f) Experimental reaction times at 1, 3, 5, 7, and 15 days, respectively.

Detailed observations from FESEM micrographs (secondary and backscattered electron images) reveal the crystalline properties and morphology of the CaP precipitates and their association with the cellulose nanofibers (Figure 3). SEM images show the BC network with crystals coating intimately the cellulose fibers and partially filling the pores of the three-dimensional structure. Interestingly, remnants of dead bacteria (*Komagataeibacter* sp. SU12) can also be observed forming nanofibers protruding laterally from their elongated bodies (Figure 3a: white arrows). Notably, no precipitation occurs on the surface of these bacterial remnants.

**Figure 3:**
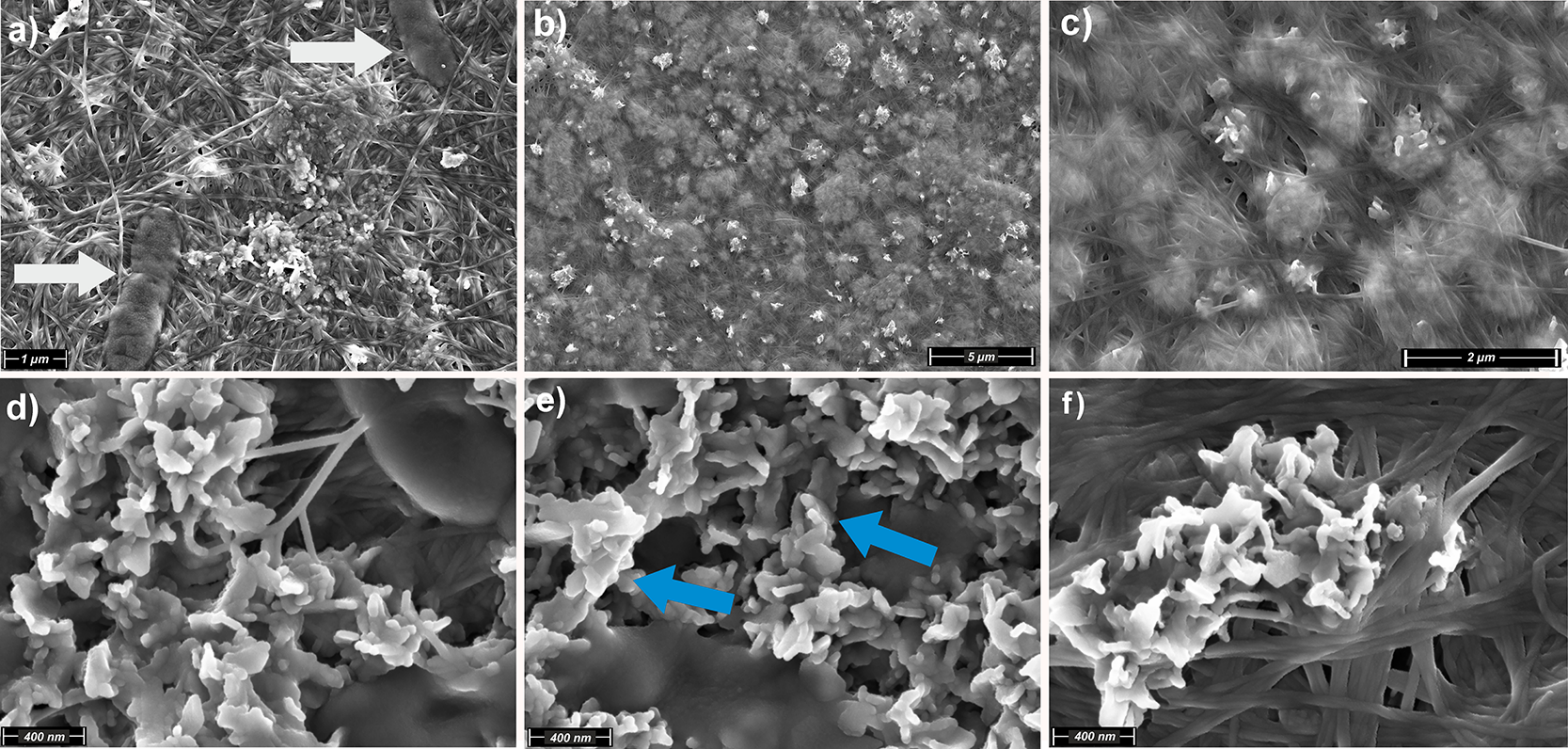
FESEM images of BC-CaP coatings: (a) BC-CaP coatings showing *Komagataeibacter* sp. SU12 remnants (white arrows); (b, c) Heterogeneous distribution of CaP aggregates embedded within the BC matrix (BSE images); (d, e) CaP crystals intimately coating the BC nanofibers (blue arrows); (f) CaP crystals forming large flower-like aggregates.

The CaP mineralization is heterogeneously distributed and embedded within the BC matrix. CaP precipitation occurs from the interior to the exterior of the BC matrix on the outer surface, forming aggregates that appear to be embedded within its thickness and progressively emerge toward the BC membrane (Figure 3b,c; BSE images). Higher-resolution images reveal crystal formation intimately coating the cellulose nanofibers (Figure 3d,e). The interaction phenomena between organic–inorganic components influence the crystal nucleation and growth during the reaction of the Ca and P-bearing solutions with the polymeric matrix of the BC nanofibers surface (Figure 3e: blue arrows). The morphology of the crystals exhibits the typical apatite plate-like shape elongated along the *c*-axis, forming larger flower-like aggregates (Figure 3f). The more isolated crystals in Figure 3d–f display an average length of 270 ± 5 nm, and an average width of 40 ± 3 nm (around 20 measurements per dimension).

EDX analyses confirmed the heterogeneous distribution of mineral formation on the cellulose matrix. Figure 4 shows the elemental mapping (calcium, phosphorus, and oxygen distribution) of the BC-CaP sample after 7 days, wherein the Ca and P atoms are highlighted (Figure 4b,c, respectively). Crystals are distributed irregularly, forming crystalline aggregates of varying sizes. Figure 5 shows one of these crystalline aggregates protruding from the BC matrix, along with its EDX spectrum. The EDX values resulted in a Ca/P ratio of around 1.75, which is slightly above 1.67, the stoichiometric hydroxyapatite ratio, the thermodynamically most stable phase among various CaP forms (e.g., brushite, monetite, OCP, TCP, or ACP).^56^ This higher Ca/P ratio can be mainly explained by the presence of CO_3_^2–^ substituting PO_4_^3–^ groups in the apatite structure (B-type substitutions). These results are consistent with the crystalline identification observed from XRD analyses.

**Figure 4:**
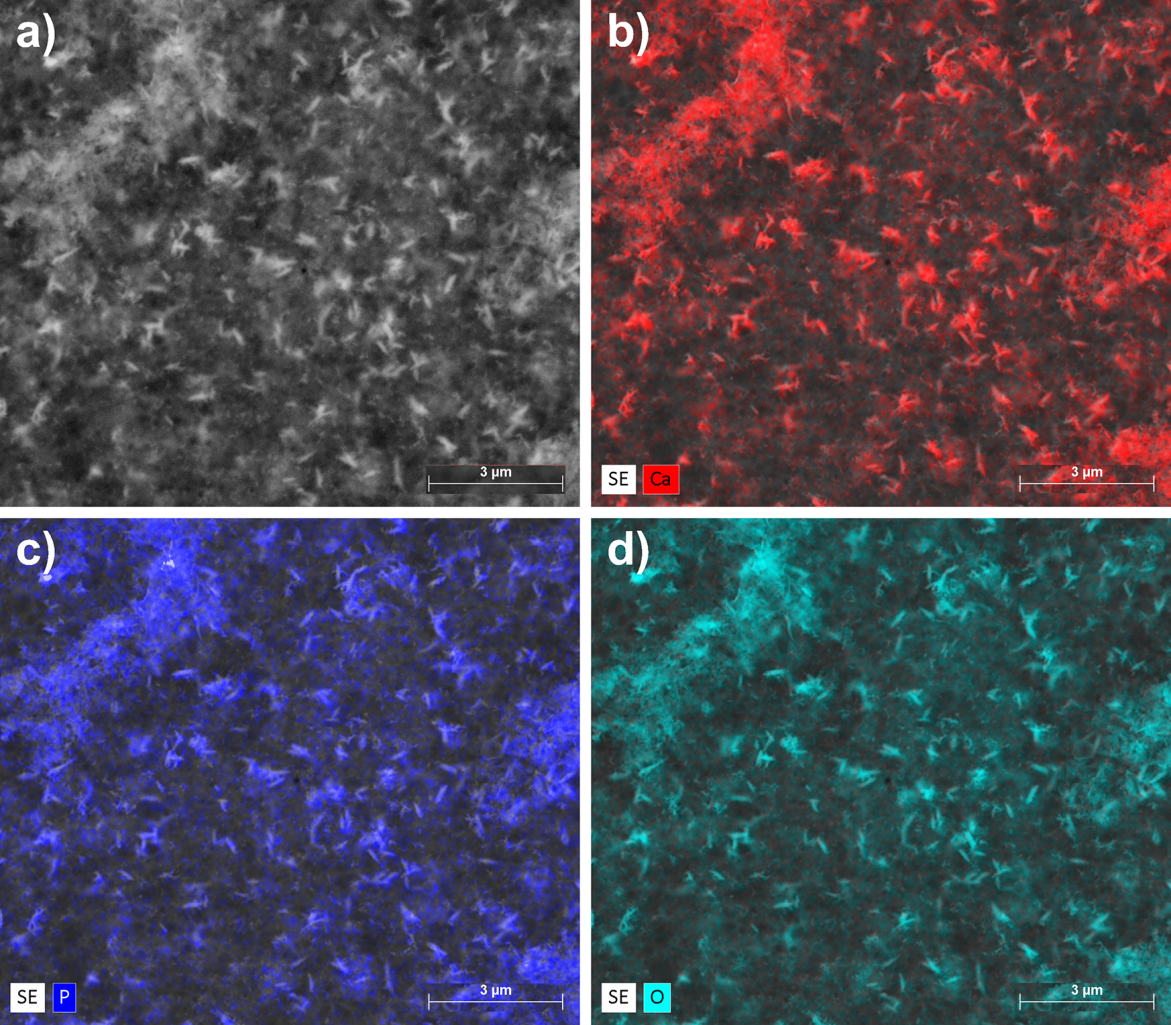
SEM micrograph and elemental distribution maps of BC-CaP surfaces obtained by EDX mapping. (a) SEM image of BC-CaP surface; (b) Elemental map showing Calcium (Ca, in red); (c) Phosphorus (P, in purple); and (d) Oxygen (O; green).

**Figure 5:**
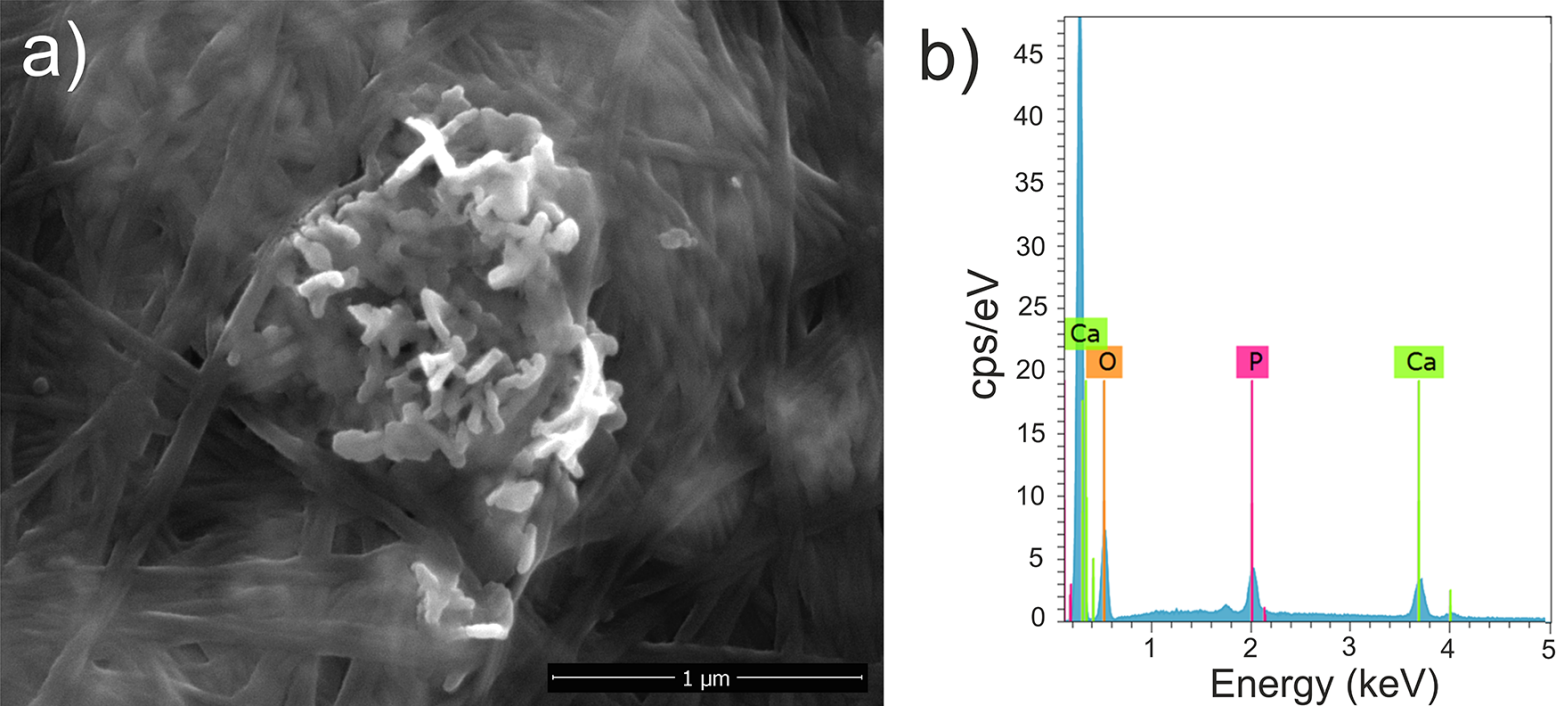
Identification of CaP aggregate on the BC matrix (a) SEM micrograph showing CaP morphology, and (b) the corresponding EDX spectrum.

The crystalline CaP phase was characterized by 2D-XRD pattern integration (i.e., unidimensional 2θ scan). Figure 6 shows the XRD results of the BC surface and mineralized BC-Ap 15 days samples. The broad diffraction peaks reflect the nanocrystalline character of both the BC substrate and CaP precipitation. The XRD pattern for BC (Figure 6a) shows diffraction peaks at 2θ angles of approximately 14.5, 16.6, and 22.6°, corresponding to the crystalline cellulose I structure planes (101), (10 $\overset{-}{1}$ ) and (002), respectively.^57^ On the other hand, the XRD pattern for BC-Ap 15 days sample (Figure 6b) corresponds to hydroxyapatite, as identified by comparison with the standard JCPDS card No. 01-074-0565 (International Centre for Diffraction Data). Specifically, the diffraction peak located at 25.9° corresponds to the (002) diffraction line (*c-axis* direction), while a broad band in the 2θ range of 32–34° to the combined (121), (211), (112), and (300) diffraction lines. This phase identification, along with similar crystalline properties, was observed consistently for all experimental reaction times. The 2D-XRD patterns (Figure 6, insets) show continuous diffraction arcs within the Debye–Scherrer ring, indicating a crystal random orientation of the crystalline phases. The broadness of the diffraction peaks points to the low degree of crystallinity of the BC and Ap phases presented in the samples, which correlates with the nanometer scale of the crystals, as previously observed in the SEM analysis.

**Figure 6:**
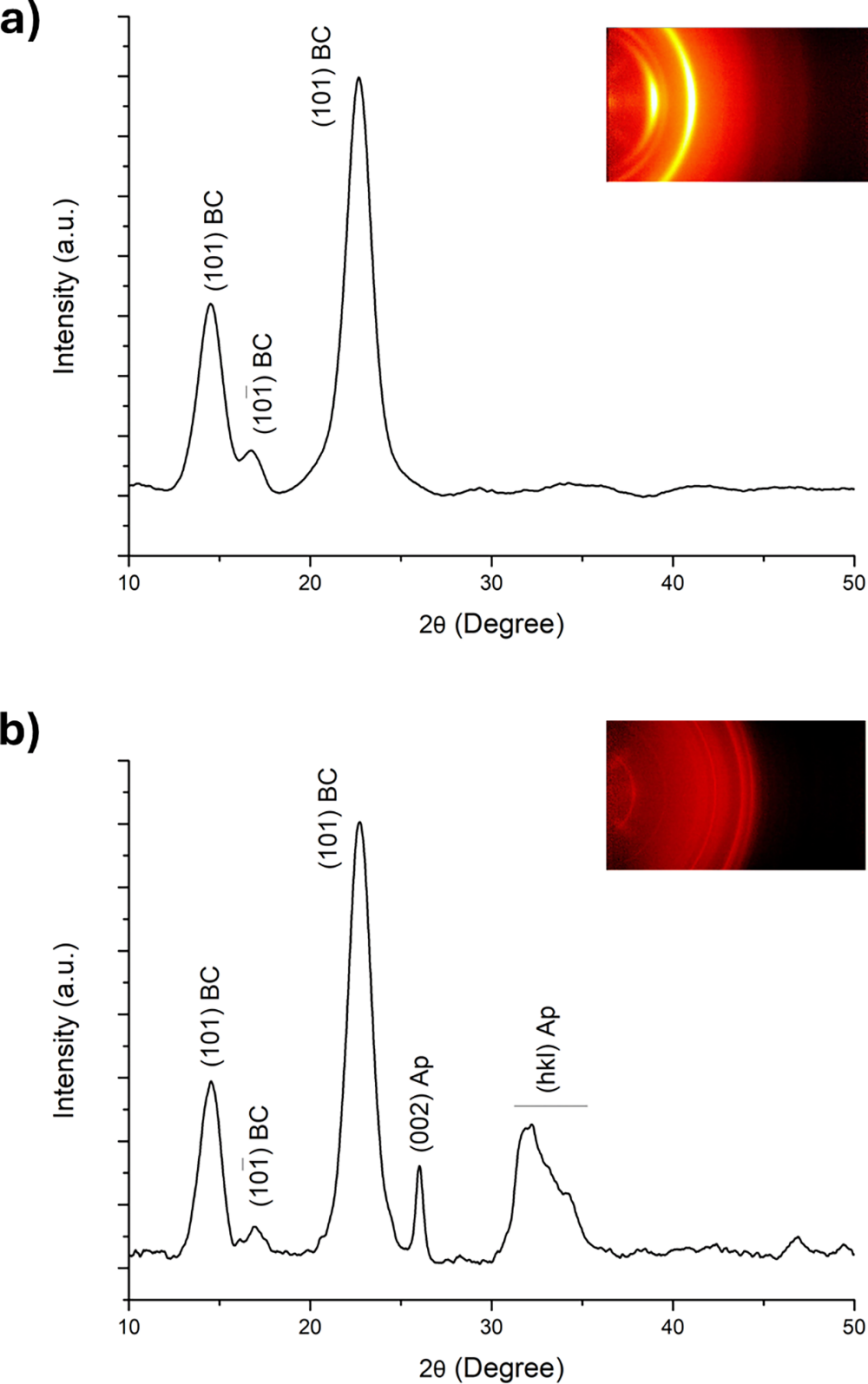
XRD analysis of (a) bacterial cellulose (BC) surface, and (b) BC-Ap 15 days samples (Ap; apatite). Insets display the 2D-XRD patterns for each sample, used to obtain the unidimensional integration scan (2θ degree).

Vibrational spectroscopies allow us to identify the molecular composition of the structural organic groups of BC and the molecular vibration modes associated with Ap.^18,34^ Detailed vibrational band assignments for BC and BC-Ap samples in the ATR-FTIR and Raman spectra are provided in Figure 7 and Tables 1 and 2 (ATR-FTIR and Raman band assignments, respectively). The ATR-FTIR spectrum of BC (Figure 7a) shows an intense narrow band at 1030 cm^–1^ corresponding to C–O stretching vibrations associated with the cyclic glucose of cellulose. Additional bands at 1160 and 897 cm^–1^ indicate β-1,4-glycosidic linkages.^58–65^ Other bands observed in the 400–700 cm^–1^ range (δCOH out of plane), at 1650 cm^–1^ (hydrogen-bonded), and at 3340 cm^–1^ (γOH covalent bond) were also identified.^10,54,58,59,61–63,65^ These cellulose biomolecules engage in electrostatic interactions between the functional groups of BC (e.g., carboxylate and hydroxyl groups) and calcium ions, promoting the formation of apatite crystals.^66^ The FTIR spectrum of the BC-Ap samples (Figure 7b), showed intense bands at 960–1103 cm^–1^ (ν_1_-ν_3_ PO_4_^3–^) and 557 cm^–1^ (ν_4_ PO_4_^3–^), corresponding to characteristic apatite IR absorption bands.^38,67–72^ Other less intense bands are intended at 434 cm^–1^ (ν_2_ PO_4_^3–^) and 1556 cm^–1^ (ν_3_ CO_3_).^68,70,72^ However, the ATR-FTIR analysis faced challenges in precisely identifying BC-Ap interactions due to overlapping signals from BC groups and Ap modes.

**Figure 7:**
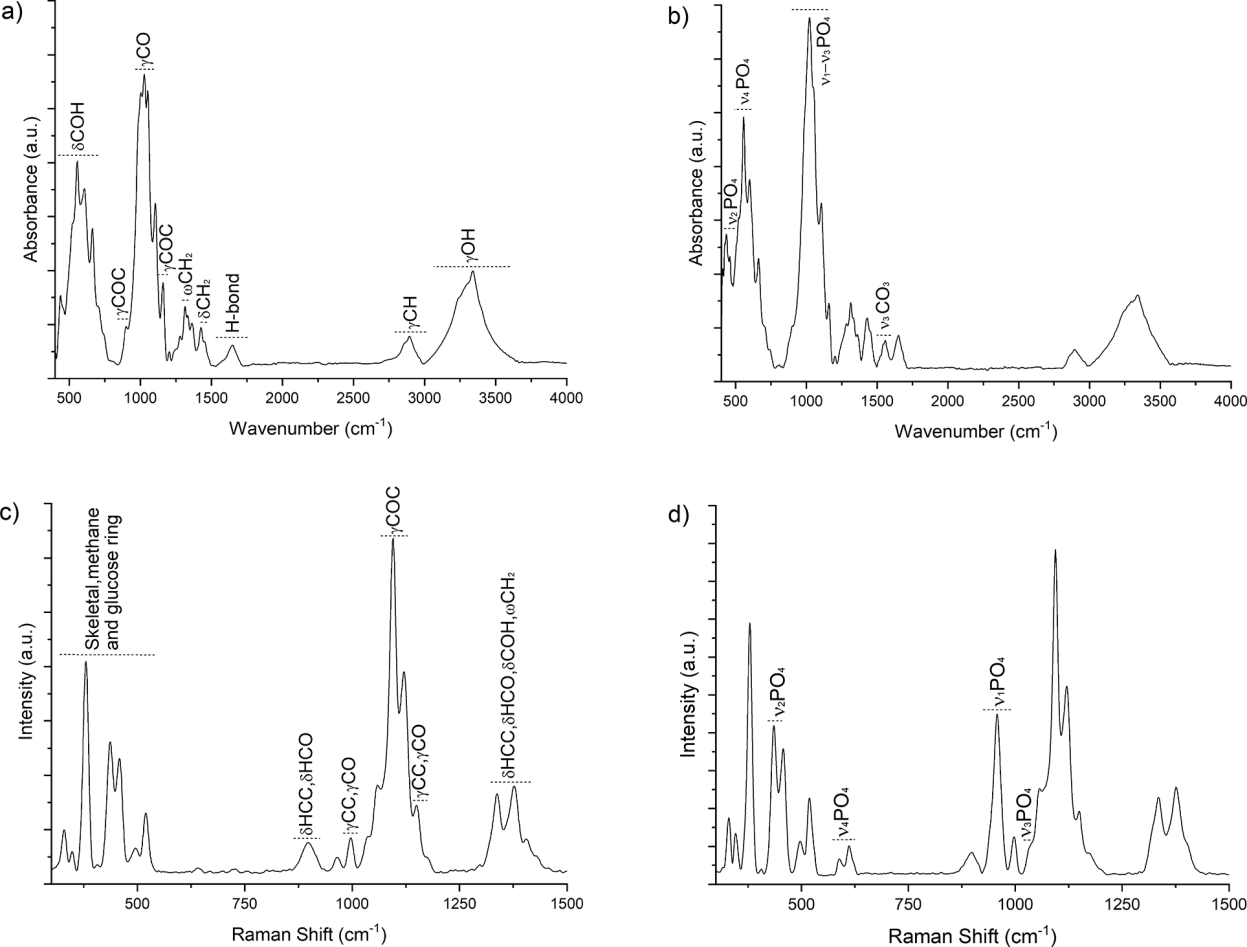
Vibrational analysis of BC-Ap 15-day sample using ATR-FTIR and Raman spectroscopy. (a) ATR-FTIR spectrum of BC, indicating OH, COH, COC, CO, and CH bands (organic modes), (b) ATR-FTIR spectra of CaP, highlighting the vibrational modes of phosphate ν_1_–ν_3_ PO_4_^3–^ and carbonate ν_3_ CO_3_ groups (inorganic modes), (c) Raman spectra of BC, indicating skeletal, methane, glucose ring, COC, and CH bands, (d) Raman spectra of CaP highlighting the vibrational modes of phosphate groups ν_1_–ν_4_ PO_4_^3–^.

**Table 1:** ATR-FTIR Spectra Vibrational Band Assignments Observed in Bacterial Cellulose (BC) and Apatite (Ap) Components

**band position (cm^–1^)**	**BC band assignments**	**reference**
400–700	δCOH out of a plane	^10,59^
897	γCOC at β-glycosidic linkage	^58,59,62,63,65^
1030	γCO at C-6	^59,61,63^
1105	ring in-plane	^59,62,64^
1160	γCOC at β-glycosidic linkage	^58–61,63,64^
1315	ωCH_2_ at C-6	^59,61–64^
1425	δCH_2_ at C-6	^59–61,64^
1650	OH groups	^10,58,59^
2894	γCH of CH_2_ and CH_3_	^10,54,58,59,62–64^
3340	γOH covalent bond	^54,58,59,61–65^

**band position (cm^–1^)**	**Ap band assignments**	**reference**
434	ν_2_ PO_4_^3–^	^72^
557	ν_4_ PO_4_^3–^	^38,67,70,72^
960	ν_1_ PO_4_^3–^	^38,67,68,70–72^
1020	ν_3_ PO_4_^3–^	^38,67,69,72^
1103	ν_3_ PO_4_^3–^	^38,67,68,72^
1556	ν_3_ CO_3_	^68,70,72^

**Table 2:** Raman Spectra Band Assignments Observed in Bacterial Cellulose (BC) and Apatite (Ap) Components

**band position (cm** ^**–1**^ **)**	**BC band assignments**	**reference**
400–550	Skeletal (δCCC, δCOC, δOCC, δOCO)Methane (δCCH, δCOH)Glucose ring (CC, CO)	^63,73,74^
897	δHCC, δHCO at glucose ring deformation	^63,73^
997	γCC, γCO	^63^
1094	γCOC skeletal	^63,73^
1120		
1152	γCC, γCO	^63,73,74^
1335	δHCC, δHCO, δCOH and ωCH_2_	^63,73,74^
1379		

**band position (cm** ^**–1**^ **)**	**Ap band assignments**	**reference**
435	ν_2_ PO_4_^3–^	^71,75,76^
590610	ν_4_ PO_4_^3–^	^70,71,75,76,78^
957	ν_1_ PO_4_^3–^	^38,70,71,75,77^
1032	ν_3_ PO_4_^3–^	^71,75^

The ATR-FTIR results show spectral characteristics of BC, such as OH, C–O, C–O–C, and C–H bonds, consistent with previous studies.^54,66^ Vibrational modes of phosphate groups (ν_1_-ν_3_ PO_4_^3–^) were also identified in the 950–1000 cm^–1^ range. It was observed that the phosphate bands overlap with the C–O and C–O–C vibrations of BC, highlighting the need for complementary spectroscopic techniques, such as Raman spectroscopy, to allow for a more detailed analysis of BC-Ap composites.

The Raman spectrum of BC (Figure 7c) reveals intense bands at 1094 and 1120 cm^–1^, corresponding to γCOC skeletal vibrations.^63,73^ Additional bands below 550 cm^–1^ are attributed to skeletal (δCCC, δCOC, δOCC, δOCO), methane (δCCH, δCOH), and glucose ring (CC, CO) vibrations.^63,73,74^ Furthermore, bands at 897 cm^–1^ (δHCC, δHCO) corresponded to glucose ring deformation; while 997 and 1152 cm^–1^ (γCC, γCO); and 1335 and 1379 cm^–1^ (δHCC, δHCO, δCOH, CH_2_) are also observed.^63,73,74^ The Raman spectrum of the BC-Ap sample (Figure 7d) confirms the presence of characteristic vibrational modes of apatite, with intense peaks at 957 cm^–1^ (ν_1_ PO_4_^3^), 435 cm^–1^ (ν_2_ PO_4_^3–^), and weaker peaks at 590 and 610 cm^–1^ (ν_4_ PO_4_^3–^), as well as 1032 cm^–1^ (ν_3_ PO_4_^3–^).^70,71,75–78^

The crystallization mechanism underlying the formation of the BC-Ap composite is directly influenced by the interfacial binding strength between the organic (BC) and inorganic (Ap) components.^79^ This interfacial affinity is determined by the intermolecular interactions occurring at the organic–inorganic phase boundaries. The structural conformation of anhydro-glucose units (AGUs) in nanocellulose consists of linear chains of β-d-glucopyranose units arranged in ribbon-like shapes and stabilized by intra- and intermolecular hydrogen bonds, where carboxyl functional groups can be selectively anchored at the surface-exposed C6 positions of the AGUs.^80,81^ Due to the structural configuration of the BC surface, hydroxyl and carboxyl functional groups can readily act as effective nucleation sites for Ap formation. This process suggests a first nucleation mechanism in which Ap crystals form through the initial adsorption of calcium ions, mediated by electrostatic interactions and complexation with negatively charged surface BC groups, followed by the subsequent binding of phosphate ions from the surrounding fluid. Therefore, the formation of BC-Oδ- BC–Oδ^–^···Ca^2+^ or BC–COO^–^···Ca^2+^ coordination complexes on the surface of BC nanofibrils serves as a critical prerequisite for the nucleation of the calcium phosphate (CaP) precursor phase, which subsequently evolves into apatite through the sustained uptake of phosphate ions from the solution. Furthermore, previous molecular modeling studies have revealed that interfacial structures stabilized by electrostatic interactions between BC and CaP crystalline surfaces promote the formation of plate-like hydroxyapatite morphologies,^66^ as further evidenced by SEM imaging in the current study.

Thermogravimetric analyses (Figure 8) of the BC and BC-Ap 15d samples show distinct thermal decomposition patterns and residual mass differences after heat treatment up to 900 °C. At temperatures below 200 °C, there is no significant weight loss (i.e., <2% for BC) due to moisture and volatile compounds, remaining stable until higher temperatures. Around 320 °C–350 °C, significant weight loss occurs due to BC degradation, including depolymerization, decomposition of glucosyl units, and formation of charred residue.^33^ A carbonaceous residue was observed in the pure BC sample, which remained stable from 450 °C until the end of the thermal degradation temperature. The BC-Ap exhibited a higher residue content above the latter temperature, confirming mineral deposition in the BC structure that increases with reaction time. Table 3 shows the percentages of residual mass at the end of the heat treatment (up to 900 °C), showing a difference between 3.25% for pure BC and 18.23% for the sample reacted for 15 days (i.e., indicating mineralization of approximately 15% for the BC-AP 15d reaction time). During the entire experimental reaction time, the amount of precipitate was progressive, distributing heterogeneously on the surface of the cellulose (as also observed in the SEM images).

**Figure 8:**
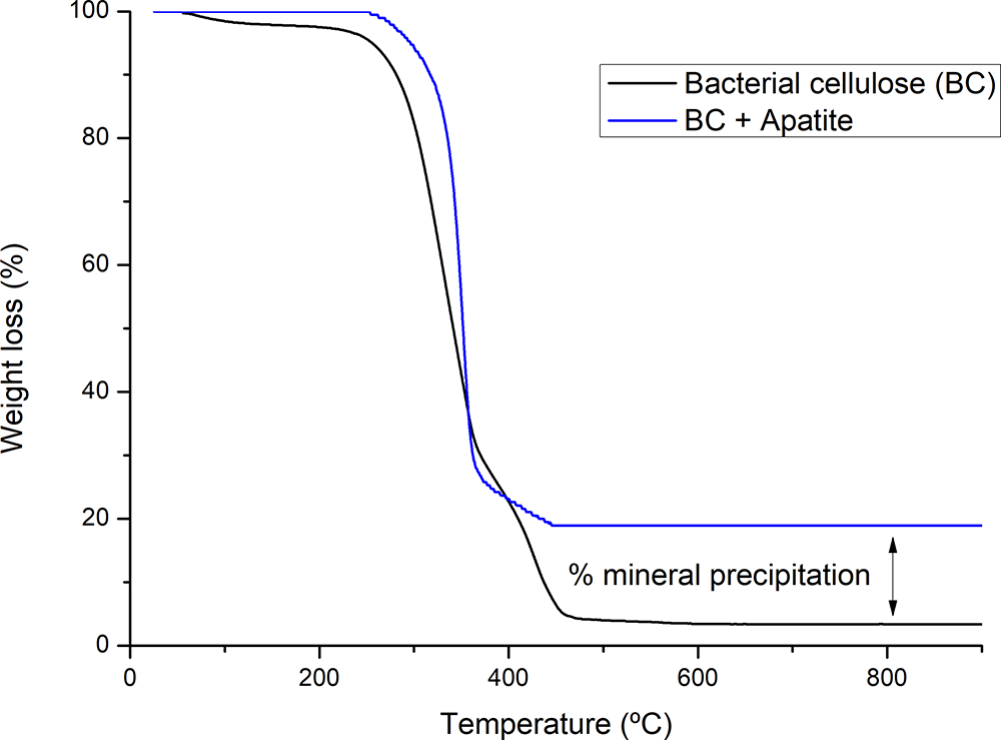
Thermogravimetric analysis (TGA) conducted in the temperature range from 25 to 900 °C using a linear heating rate of 10 °C/min. The plot shows the weight loss (%) curves of BC and BC-Ap 15d indicating the residual mass difference (double arrow line) corresponding to mineral precipitation.

**Table 3:** Thermogravimetric Analysis (TGA) Results from Residual Mass (%) of BC-Ap Samples under Different Experimental Times

**sample**	**residual mass (%)**
BC	3.25 ± 0.19
BC-Ap 1d	5.05 ± 1.59
BC-Ap 3d	9.58 ± 0.89
BC-Ap 5d	11.61 ± 0.48
BC-Ap 7d	13.85 ± 0.89
BC-Ap 15d	18.23 ± 1.23

In the present research, we have proved that the SDVD technique allows for coating one of the two surfaces of the BC membrane, yielding a hybrid material with potential bifunctional properties, i.e. osteoinductive on the apatite-coated side and barrier against cells on the opposite side. The potential application of BC-Ap composite obtained through controlled crystallization remains to be addressed, particularly concerning key material properties, such as mechanical testing and hydrophilicity/wettability. Additionally, future investigations will include cellular assays to evaluate bioactivity, cytotoxicity, and biocompatibility, although some of these characteristics have already been reported in previous studies for analogous BC-CaP materials.^38,82–84^ In any case, the control of the mineral surface formation through SDVD allows for detailed modulation of the properties of this biohybrid BC-Ap, tailoring it to specific needs depending on the type of calcified tissue and the intended application (i.e., pulp capping, guided bone regeneration). Regarding the potential scalability of BC-Ap for industrial or clinical applications, several limitations and challenges should be considered. Notably, (1) producing BC using an organic substrate derived from plant waste, rather than a standardized Hestrin-Schramm medium, compromises control over bacterial growth and influences key properties of the resulting cellulose, such as porosity and thickness. Additionally, (2) the SDVD crystallization method involves the use of microdroplets (40 μL) and extended processing times (up to 15 days) to achieve complete coating of the BC membranes, which may hinder its feasibility for large-scale or time-sensitive applications. To address the volume issue, it would be necessary to implement vapor diffusion in high volume desiccators or using a higher amount of crystallization mushrooms to multiply the number of droplets. In addition, the optimization or acceleration of the crystallization by adjusting different experimental parameters, such as reaction temperature or ionic strength (e.g., by introducing additional ions into the solution), could help reduce processing time while maintaining control over Ap crystal formation on the BC nanofibers. These factors are critical to ensure the reproducibility and scalability of BC-Ap production from laboratory to industrial production.

## 4 Conclusions

The SDVD method successfully achieved apatite (Ap) mineralization in bacterial cellulose (BC) surface, allowing precise control over precipitation. This approach resulted in a BC-Ap hybrid material, where the mineral phase presents chemical and structural characteristics similar to nanocrystalline apatite. Additionally, mineral content increased over experimental reaction times ranging from 1 to 15 days. Morphological and spectroscopic studies revealed the interaction of Ap crystals along the nanocellulose fibers. The BC-Ap composite exhibits significant potential for tissue engineering applications. Moreover, using mango waste as a substrate for BC production provides a sustainable and cost-effective solution, contributing to the development of materials from green and biocompatible technological solutions.

## Data Availability

Data will be made available on request.

## References

[1] Ahn S., Shin Y., Kim S., Jeong S., Jeong J.-O., Park J.-S., Gwon H.-J., Seo D., Nho Y.-C., Kang S., Kim C.-Y., Huh J.-B., Lim Y.-M. (2015). Characterization
of Hydroxyapatite-Coated Bacterial Cellulose Scaffold for Bone Tissue
Engineering. Biotechnol. Bioprocess Eng..

[2] Liu W., Du H., Zhang M., Liu K., Liu H., Xie H., Zhang X., Si C. (2020). Bacterial
Cellulose-Based Composite
Scaffolds for Biomedical Applications: A Review. ACS Sustainable Chem. Eng..

[3] Pértile R., Moreira S., Andrade F., Domingues L., Gama M. (2012). Bacterial Cellulose Modified Using
Recombinant Proteins to Improve
Neuronal and Mesenchymal Cell Adhesion. Biotechnol.
Prog..

[4] Rajwade J., Paknikar K., Kumbhar J. (2015). Applications of Bacterial Cellulose
and Its Composites in Biomedicine. Appl. Microbiol.
Biotechnol..

[5] Brown R., Willison J., Richardson C. (1976). Cellulose
Biosynthesis in Acetobacter
Xylinum: Visualization of the Site of Synthesis and Direct Measurement
of the in Vivo Process. Proc. Natl. Acad. Sci.
U.S.A..

[6] Wang J., Tavakoli J., Tang Y. (2019). Bacterial Cellulose Production, Properties
and Applications with Different Culture Methods–A Review. Carbohydr. Polym..

[7] Nascimento F., Torres C., Freitas F., Reis M., Crespo M. (2021). Caracterización
Funcional y Genómica de Komagataeibacter Uvaceti FXV3, Una
Bacteria Resistente al Estrés Múltiple Que Produce Un
Aumento de Los Niveles de Celulosa. Biotechnol.
Rep..

[8] Machado R., Gutierrez J., Tercjak A., Trovatti E., Yahib F., Moreno G., Nascimento A., Berreta A., Ribeire S., Barud H. (2016). Komagataeibacter
Rhaeticus as an Alternative Bacteria for Cellulose
Production. Carbohydr. Polym..

[9] Quijano L., Rodrigues R., Fischer D., Tovar-Castro J., Payne A., Navone L., Hu Y., Yan H., Pinmanee P., Poon E., Yang J.-H., Barro E. (2024). Bacterial
Cellulose Cookbook: A Systematic Review on Sustainable and Cost-Effective
Substrates. J. Bioresour. Bioprod..

[10] Calderón-Toledo S., Horue M., Alvarez V., Castro G., Zavaleta A. (2022). Isolation
and Partial Characterization of Komagataeibacter Sp. SU12 and Optimization
of Bacterial Cellulose Production Using Mangifera Indica Extracts. J. Chem. Technol. Biotechnol..

[11] Mardawati E., Rahmah D., Rachmadona N., Saharina E., Pertiwi T., Zahrad S., Ramdhani W., Srikandace Y., Ratnaningrum D., Endah E., Andriani D., Khoo K., Pasaribu K., Satoto R., Karina M. (2023). Pineapple
Core from
the Canning Industrial Waste for Bacterial Cellulose Production by
Komagataeibacter Xylinus. Heliyon.

[12] Moukamnerd C., Ounmuang K., Konboa N., Insomphun C. (2020). Bacterial
Cellulose Production by Komagataeibacter Nataicola TISTR 2661 by Agro-Waste
as a Carbon Source. Chiang Mai J. Sci..

[13] Pasteris J., Wopenka B., Valsami-Jones E. (2008). Bone and Tooth
Mineralization: Why
Apatite?. Elements.

[14] Zapanta R. (1981). Apatites in
Biological Systems. Prog. Cryst. Growth Charact.
Mater..

[15] Gómez-Morales J., Iafisco M., Delgado-López J. M., Sarda S., Drouet C. (2013). Progress on
the Preparation of Nanocrystalline Apatites
and Surface Characterization: Overview of Fundamental and Applied
Aspects. Progress in Crystal Growth and Characterization
of Materials.

[16] Akazawa H. (2022). Characterization
of Crystallographic Orientation and Lattice Disorder in Hydroxyapatite
Thin Films by Raman Scattering. Ceram. Int..

[17] Mohd N., Koshy P., Abdullah H., Idris M., Lee T. (2019). Syntheses
of Hydroxyapatite from Natural Sources. Heliyon.

[18] Liu Q., Huang S., Matinlinna J., Chen Z., Pan H. (2013). Insight into
Biological Apatite: Physiochemical Properties and Preparation Approaches. Biomed. Res. Int..

[19] Baskar K., Saravana B., Gurucharan I., Mahalaxmi S., Rajkumar G., Dhivya V., Kishen A. (2022). Eggshell Derived
Nano-Hydroxyapatite
Incorporated Carboxymethyl Chitosan Scaffold for Dentine Regeneration:
A Laboratory Investigation. Int. Endod. J..

[20] Duarte E., Chagas B., Andrade F., Brígida A., Borges M., Muniz C., Filho M., Saraiva J., Feitosa J., Rosa M. (2015). Production of Hydroxyapatite–Bacterial
Cellulose Nanocomposites from Agroindustrial Wastes. Cellulose.

[21] Grande C., Torres F., Gomez C., Baño C. (2009). Nanocomposites
of Bacterial Cellulose/Hydroxyapatite for Biomedical Applications. Acta Biomater..

[22] Ingole V., Vuherer T., Maver U., Vinchurkar A., Ghule A., Kokol V. (2020). Mechanical Properties and Cytotoxicity
of Differently Structured Nanocellulose-Hydroxyapatite Based Composites
for Bone Regeneration Application. Nanomaterials.

[23] Mondal S., Park S., Choi J., Vu T., Doan V., Vo T., Lee B., Oh J. (2023). Hydroxyapatite:
A Journey from Biomaterials
to Advanced Functional Materials. Adv. Colloid
Interface Sci..

[24] Wang X., Yang X., Xiao X., Li X., Chen C., Sun D. (2024). Biomimetic Design of Platelet-Rich
Plasma Controlled Release Bacterial
Cellulose/Hydroxyapatite Composite Hydrogel for Bone Tissue Engineering. Int. J. Biol. Macromol..

[25] Raut M., Asare E., Syed S., Amadi E., Roy I. (2023). Bacterial
Cellulose-Based Blends and Composites: Versatile Biomaterials for
Tissue Engineering Applications. Int. J. Mol.
Sci..

[26] Wan Y., Huang Y., Yuan C. (2007). Biomimetic
Synthesis of Hydroxyapatite/Bacterial
Cellulose Nanocomposites for Biomedical Applications. Mater. Sci. Eng., C.

[27] Huang Y., Wang J., Yang F., Shao Y., Zhang X., Dai K. (2017). Modification and Evaluation of Micro-Nano
Structured Porous Bacterial
Cellulose Scaffold for Bone Tissue Engineering. Mater. Sci. Eng., C.

[28] Reina N., Laffosse J. (2014). Biomecánica Del Hueso: Aplicación
al
Tratamiento y a La Consolidación de Las Fracturas. EMC - Aparato Locomotor.

[29] Khan S., Ul-Islam M., Ullah M. W., Zhu Y., Narayanan K. B., Han S. S., Park J. K. (2022). Fabrication Strategies
and Biomedical
Applications of Three-Dimensional Bacterial Cellulose-Based Scaffolds:
A Review. Int. J. Biol. Macromol..

[30] Lamboni L., Xu C., Clasohm J., Yang J., Saumer M., Schäfer K.-H., Yang G. (2019). Silk Sericin-Enhanced Microstructured Bacterial Cellulose as Tissue
Engineering Scaffold towards Prospective Gut Repair. Mater. Sci. Eng., C.

[31] Xing C., Ge S., Huang B., Bo Y., Zhang D., Zheng Z. (2012). Biomimetic
Synthesis of Hierarchical Crystalline Hydroxyapatite Fibers in Large-Scale. Mater. Res. Bull..

[32] Arkharova N., Severin A., Khripunov A., Krasheninnikov S., Tkachenko A., Orekhov A., Davydova G., Rakova E., Klechkovskaya V. (2019). Composite Films Based on Bacterial
Cellulose and Nanocrystals
of Hydroxyapatite: Morphology, Structure, and Properties. Polym. Sci. Ser. A.

[33] Saska S., Barud H., Gaspar A., Marchetto R., Ribeiro S., Messaddeq Y. (2011). Bacterial
Cellulose-Hydroxyapatite
Nanocomposites for Bone Regeneration. Int. J.
Biomater..

[34] Yin N., Chen S., Ouyang Y., Tang L., Yang J., Wang H. (2011). Biomimetic Mineralization Synthesis of Hydroxyapatite Bacterial Cellulose
Nanocomposites. Prog. Nat. Sci..

[35] Iafisco M., Gómez-Morales J., Hernández-Hernández M., García-Ruiz J., Roveri N. (2010). Biomimetic Carbonate–Hydroxyapatite
Nanocrystals Prepared by Vapor Diffusion. Adv.
Eng. Mater..

[36] Gómez-Morales J., Verdugo-Escamilla C., Gavira J. (2016). Bioinspired Calcium Phosphate Coated
Mica Sheets by Vapor Diffusion and Its Effects on Lysozyme Assembly
and Crystallization. Cryst. Growth Des..

[37] Gómez-Morales J., González-Ramírez L., Verdugo-Escamilla C., Fernández Penas R., Oltolina F., Prat M., Falini G. (2019). Induced Nucleation of Biomimetic
Nanoapatites on Exfoliated
Graphene Biomolecule Flakes by Vapor Diffusion in Microdroplets. Crystals.

[38] Torres-Mansilla A., Álvarez-Lloret P., Voltes-Martínez A., López-Ruiz E., Baldión P., Marchal J., Gómez-Morales J. (2023). Apatite-Coated
Outer Layer Eggshell Membrane: A Novel Osteoinductive Biohybrid Composite
for Guided Bone/Tissue Regeneration. Biomater.
Adv..

[39] Torres-Mansilla A., Hincke M., Voltes A., López-Ruiz E., Baldión P. A., Marchal J. A., Álvarez-Lloret P., Gómez-Morales J. (2023). Eggshell Membrane as a Biomaterial for Bone Regeneration. Polymers (Basel).

[40] Peng B., Mohanty A., Misra M. (2020). Studies on Durability of Sustainable
Biobased Composites: A Review. RSC Adv..

[41] Thomas P., Duolikun T., Rumjit N., Moosavi S., Lai C., Bin M., Fen L. (2020). Comprehensive
Review on Nanocellulose: Recent Developments,
Challenges and Future Prospects. J. Mech. Behav.
Biomed. Mater..

[42] Zhao Y., Sun B., Wang T., Yang L., Xu X., Chen C., Wei F., Lv W., Zhang L., Sun D. (2020). Synthesis of Cellulose–Silica
Nanocomposites by in Situ Biomineralization during Fermentation. Cellulose.

[43] Sequeira S., Evtuguin D., Portugal I. (2009). Preparation and Properties
of Cellulose/Silica
Hybrid Composites. Polym. Compos..

[44] Feng J., Le D., Nguyen S., Tan V., Jewell D., Duong H. (2016). Silica-Cellulose
Hybrid Aerogels for Thermal and Acoustic Insulation Applications. Colloids Surf..

[45] Baghdad K., Hasnaoui A. (2020). Zeolite–Cellulose
Composite Membranes: Synthesis
and Applications in Metals and Bacteria Removal. J. Environ. Chem. Eng..

[46] Miller G. (1959). Use of Dinitrosalicylic
Acid Reagent for Determination of Reducing Sugar. Anal. Chem..

[47] Hestrin S., Schramm M. (1954). Synthesis of Cellulose by Acetobacter Xylinum. 2. Preparation
of Freeze-Dried Cells Capable of Polymerizing Glucose to Cellulose. Biochem. J..

[48] Naomi R., Bt R., Fauzi M. (2020). Plant- vs. Bacterial-Derived Cellulose for Wound Healing:
A Review. Int. J. Environ. Res. Public Health.

[49] Popa L., Ghica M. V., Tudoroiu E.-E., Ionescu D.-G., Dinu-Pîrvu C.-E. (2022). Bacterial
Cellulose—A Remarkable Polymer as a Source for Biomaterials
Tailoring. Materials.

[50] Gorgieva S., Trček J. (2019). Bacterial
Cellulose: Production, Modification and Perspectives
in Biomedical Applications. Nanomaterials (Basel).

[51] Barja F. (2021). Bacterial
Nanocellulose Production and Biomedical Applications. J. Biomed. Res..

[52] Molina G., Basmaji P., Costa L. M. M., dos Santos M. L., dos Santos Riccardi C., Guastaldi F. P. S., Scarel-Caminaga R. M., de Oliveira Capote T. S., Pizoni E., Guastaldi A. C. (2017). Surface
Physical Chemistry Properties in Coated Bacterial Cellulose Membranes
with Calcium Phosphate. Mater. Sci. Eng.: C.

[53] Suneetha M., Kim H., Han S. (2024). Bone-like
Apatite Formation in Biocompatible Phosphate-Crosslinked
Bacterial Cellulose-Based Hydrogels for Bone Tissue Engineering Applications. Int. J. Biol. Macromol..

[54] Bayir E., Bilgi E., Hames E., Sendemir A. (2019). Production of Hydroxyapatite–Bacterial
Cellulose Composite Scaffolds with Enhanced Pore Diameters for Bone
Tissue Engineering Applications. Cellulose.

[55] Hu J., Wu H., Liang S., Tian X., Liu K., Jiang M., Dominic C. D. M., Zhao H., Duan Y., Zhang J. (2023). Effects of
the Surface Chemical Groups of Cellulose Nanocrystals on the Vulcanization
and Mechanical Properties of Natural Rubber/Cellulose Nanocrystals
Nanocomposites. Int. J. Biol. Macromol..

[56] Dorozhkin S. (2011). Calcium Orthophosphates:
Occurrence, Properties, Biomineralization. Pathological
Calcification and Biomimetic Applications. Biomatter.

[57] Almasi H., Ghanbarzadeh B., Dehghannya J., Entezami A. A., Asl A. (2015). Novel Nanocomposites
Based on Fatty Acid Modified Cellulose Nanofibers/Poly­(Lactic Acid):
Morphological and Physical Properties. Food
Packag. Shelf Life.

[58] Avcioglu N., Birben M., Seyis I. (2021). Optimization and Physicochemical
Characterization of Enhanced Microbial Cellulose Production with a
New Kombucha Consortium. Process Biochem..

[59] Cichosz S., Masek A. (2020). IR Study on Cellulose
with the Varied Moisture Contents: Insight
into the Supramolecular Structure. Materials.

[60] Haafiz M., Hassan A., Zakaria Z., Inuwa I. (2014). Isolation and Characterization
of Cellulose Nanowhiskers from Oil Palm Biomass Microcrystalline Cellulose. Carbohydr. Polym..

[61] Kumar V., Sharma D., Bansal V., Mehta D., Sangwan R., Yadav S. (2019). Efficient and Economic Process for
the Production of Bacterial Cellulose
from Isolated Strain of Acetobacter Pasteurianus of RSV-4 Bacterium. Bioresour. Technol..

[62] Lahiri D., Nag M., Dutta B., Dey A., Sarkar T., Pati S., Edinur H., Abdul Z., Mohd N., Ray R. (2021). Bacterial
Cellulose: Production, Characterization, and Application as Antimicrobial
Agent. Int. J. Mol. Sci..

[63] Makarem M., Lee C., Kafle K., Huang S., Chae I., Yang H., Kubicki J., Kim S. (2019). Probing Cellulose Structures with
Vibrational Spectroscopy. Cellulose.

[64] Oh S., Yoo D., Shin Y., Kim H., Kim H., Chung Y., Park W., Youk J. (2005). Crystalline
Structure Analysis of
Cellulose Treated with Sodium Hydroxide and Carbon Dioxide by Means
of X-Ray Diffraction and FTIR Spectroscopy. Carbohydr. Res..

[65] Proniewicz L., Paluszkiewicz C., Wesełucha-Birczyńska A., Majcherczyk H., Barański A., Konieczna A. (2001). FT-IR and
FT-Raman Study of Hydrothermally Degradated Cellulose. J. Mol. Struct..

[66] Nge T. T., Sugiyama J. (2007). Surface Functional
Group Dependent Apatite Formation
on Bacterial Cellulose Microfibrils Network in a Simulated Body Fluid. J. Biomed. Mater. Res., Part A.

[67] Berzina-Cimdina, L. ; Borodajenko, N. Research of Calcium Phosphates Using Fourier Transform Infrared Spectroscopy. In Infrared Spectroscopy - Materials Science, Engineering and Technology; Intech, 2012; pp. 123–148.

[68] Gómez-Morales J., Verdugo-Escamilla C., Fernández-Penas R., María
Parra-Milla C., Drouet C., Maube-Bosc F., Oltolina F., Prat M., Fernández-Sánchez J. (2018). Luminescent
Biomimetic Citrate-Coated Europium-Doped Carbonated Apatite Nanoparticles
for Use in Bioimaging: Physico-Chemistry and Cytocompatibility. RSC Adv..

[69] Hammerli J., Hermann J., Tollan P., Naab F. (2021). Measuring in Situ CO2
and H2O in Apatite via ATR-FTIR. Contrib. to
Mineral. Petrol..

[70] Rehman I., Bonfield W. (1997). Characterization of
Hydroxyapatite and Carbonated Apatite
by Photo Acoustic FTIR Spectroscopy. J. Mater.
Sci. Mater. Med..

[71] Rey, C. ; Combes, C. ; Drouet, C. ; Grossin, D. ; Sarda, S. Advances in Calcium Phosphate Biomaterials. In Characterization of Calcium Phosphates Using Vibrational Spectroscopies; Ben-Nissan, B. , Ed.; Springer, 2014.

[72] Stoch A., Jastrzębski W., Brożek A., Trybalska B., Cichocińska M., Szarawara E. (1999). FTIR Monitoring of the Growth of
the Carbonate Containing Apatite Layers from Simulated and Natural
Body Fluids. J. Mol. Struct..

[73] Szymańska-Chargot M., Cybulska J., Zdunek A. (2011). Sensing the Structural Differences
in Cellulose from Apple and Bacterial Cell Wall Materials by Raman
and FT-IR Spectroscopy. Sensors.

[74] Wiley J., Atalla R. (1987). Band Assignments in
the Raman Spectra of Celluloses. Carbohydr.
Res..

[75] Penel G., Leroy G., Rey C., Bres E. (1998). MicroRaman Spectral
Study of the PO4 and CO3 Vibrational Modes in Synthetic and Biological
Apatites. Calcif. Tissue Int..

[76] Tsuda H., Arends J. (1997). Raman Spectroscopy
in Dental Research: A Short Review
of Recent Studies. Adv. Dent. Res..

[77] Walters M., Leung Y., Blumenthal N., Konsker K., LeGeros R. (1990). A Raman and
Infrared Spectroscopic Investigation of Biological Hydroxyapatite. J. Inorg. Biochem..

[78] Xu J., Butler I., Gilson D. (1999). FT-Raman and
High-Pressure Infrared
Spectroscopic Studies of Dicalcium Phosphate Dihydrate (CaHPO4.2H2O)
and Anhydrous Dicalcium Phosphate (CaHPO4). Spectrochim. Acta A Mol. Biomol. Spectrosc..

[79] Tolmachev D. A., Lukasheva N. V. (2012). Interactions
Binding Mineral and Organic Phases in
Nanocomposites Based on Bacterial Cellulose and Calcium Phosphates. Langmuir.

[80] Montanari S., Roumani M., Heux L., Vignon M. R. (2005). Topochemistry of
Carboxylated Cellulose Nanocrystals Resulting from TEMPO-Mediated
Oxidation. Macromolecules.

[81] Channab B.-E., El Idrissi A., Essamlali Y., Zahouily M. (2024). Nanocellulose: Structure,
Modification, Biodegradation and Applications in Agriculture as Slow/Controlled
Release Fertilizer, Superabsorbent, and Crop Protection: A Review. Journal of Environmental Management.

[82] Kohli N., Sharma V., Orera A., Sawadkar P., Owji N., Frost O., Bailey R., Snow M., Knowles J., Blunn G., García-Gareta E. (2021). Pro-Angiogenic
and
Osteogenic Composite Scaffolds of Fibrin, Alginate and Calcium Phosphate
for Bone Tissue Engineering. J. Tissue Eng..

[83] Mazzoni E., D’Agostino A., Manfrini M., Maniero S., Puozzo A., Bassi E., Marsico S., Fortini C., Trevisiol L., Patergnani S., Tognon M. (2017). Human Adipose Stem Cells Induced
to Osteogenic Differentiation by an Innovative Collagen/Hydroxylapatite
Hybrid Scaffold. FASEB J..

[84] Patil T., Saha S., Biswas A. (2017). Preparation
and Characterization
of HAp Coated Chitosan-Alginate PEC Porous Scaffold for Bone Tissue
Engineering. Macromol. Symp..

